# Machine metaphors and ethics in synthetic biology

**DOI:** 10.1186/s40504-018-0077-y

**Published:** 2018-06-04

**Authors:** Joachim Boldt

**Affiliations:** Department of Medical Ethics and the History of Medicine, University of Freiburg, Freiburg, Germany

**Keywords:** Metaphor, Ethics, Bioethics, Synthetic biology, Biosafety, Means-end relation, Techno-fix, Theory of science

## Abstract

The extent to which machine metaphors are used in synthetic biology is striking. These metaphors contain a specific perspective on organisms as well as on scientific and technological progress. Expressions such as “genetically engineered machine”, “genetic circuit”, and “platform organism”, taken from the realms of electronic engineering, car manufacturing, and information technology, highlight specific aspects of the functioning of living beings while at the same time hiding others, such as evolutionary change and interdependencies in ecosystems. Since these latter aspects are relevant for, for example, risk evaluation of uncontained uses of synthetic organisms, it is ethically imperative to resist the thrust of machine metaphors in this respect. In addition, from the perspective of the machine metaphor viewing an entity as a moral agent or patient becomes dubious. If one were to regard living beings, including humans, as machines, it becomes difficult to justify ascriptions of moral status. Finally, the machine metaphor reinforces beliefs in the potential of synthetic biology to play a decisive role in solving societal problems, and downplays the role of alternative technological, and social and political measures.

## Introduction

An important part of the moral challenges of genetic engineering originates from misunderstandings fostered by the use of metaphors, asserts philosopher Peter Janich (Janich 2001). What Janich writes with regard to the genetic information metaphor holds true for synthetic biology’s machine metaphors as well. The machine metaphor is a central part of a specific understanding of what synthetic biology is. It is both an expression of this understanding of what synthetic biology is and it shapes synthetic biology research accordingly, as well (Hellsten and Nerlich 2011, Vincent 2016).

For one thing, metaphors contain ways of thinking about the ontology of one’s object of research and about the nature of one’s research activity itself. For example, with regard to synthetic biology whether or not the set of ontological characteristics suggested by the machine metaphor captures adequately what an organism, as a matter of fact, is, and how it can be described and explained is the subject of recurring dispute (Boudry and Pigliucci 2013, Nicholson 2013, Rosen 1993). For another thing, ontological assumptions of this kind have ethical implications regarding one’s perception of safety issues, regarding what one thinks of as justified ways to act and react to the object, and regarding what one assumes a technology to be capable of. In what is to follow, some of the ontological assumptions of the machine metaphor will be identified and analyzed. Whether or not these assumptions adequately capture what an organism is, will not be at the center of the argument, though. Instead, the argument will focus on the latter ethical implications that a machine metaphor inspired understanding of synthetic biology and its research objects may have. As a result, it will not argued here that the machine metaphor should be rejected tout court. The machine metaphor may for example help to look for and identify controllable causes of molecular intracellular processes, and engineering these regularities may help to develop single cell organisms with valuable novel traits. It will be argued, though, that one needs to keep in mind that the machine metaphor is a metaphor and as such captures at best parts of the characteristics of organisms. One should be aware that this mismatch can influence what one expects organisms to be able to do, and how one behaves towards them, in ethically relevant ways.

## The machine metaphor and its implications

In synthetic biology, to name but a few examples, single cell organisms are described as “genetically engineered machines” (iGEM Foundation n.d.), intracellular molecular processes are “genetic circuits” (Weiss et al. 2003), and genetically engineered organisms are referred to as “platform organisms” (Roelants et al. 2013). Obviously, synthetic biology makes use of the metaphor of the machine and its variants, most notably its information technology equivalent, the computer, as a central conceptual lens to describe, explain and modify molecular intra- and intercellular processes.

Applying machine metaphors in order to describe and understand natural objects and processes is not a new phenomenon. In the seventeenth century, the mechanical clock was used to this end, in the eighteenth century the balance was an influential metaphor, in the nineteenth century the steam engine metaphor appeared, and in the twentieth century and up to day the computer, the information processing machine, replaced these older machine metaphors (Lunteren 2016, Zaccaria, Dedrick, and Momeni 2017). All of these machines are artefacts designed by humans, and their structural and material make-up serves as a means to attain a predefined state of affairs and thus fulfills a specific purpose for humans. Given the fact that living beings are not designed by humans, and their make-up, according to evolutionary theory, cannot be explained by reference to a specific purpose it is meant to fulfill, the widespread use of machine metaphors in modern science might come as a surprise. At the same time, if one succeeded in explaining organisms or parts of organisms *as if* they were designed to fulfill a specific purpose, this is to say that one succeeded in identifying causal relations in organisms that *allows for* their purposeful redesign and reengineering. In this sense, machine metaphors fit neatly into the modern concept of the natural sciences according to which if one understands an object, one should, in principle, be able to build it, and successfully building an object counts as an experiment that supports the theoretical hypotheses that went into its design (Köchy 2012).

Against this backdrop, the widespread use of machine metaphors in synthetic biology can be seen as an expression of the vigor with which synthetic biology introduces rational design and building ideals into biology. The engineering methodology figures prominently in synthetic biology’s multi-disciplinary background of research approaches. Biology, chemistry, physics, IT and engineering all are part of this background, with engineering approaches occupying center stage (Heinemann and Panke 2006, Schyfter 2013). Engineering principles and approaches, such as designing, modularizing, and standardizing suggest using the machine metaphor for its objects of intervention.

In addition, the machine metaphor fits neatly into a larger story of what synthetic biology is and what it is aiming at. Following this story, synthetic biology constitutes the latest step along the line of developing scientific bottom-up explanations of macro-objects and their behavior (Church and Regis 2012). At the lowest level, physics analyzes the movement and structure of atoms by identifying and analyzing subatomic structures and parts. At the next level, chemistry analyzes complex molecules by scrutinizing the simpler molecules and atoms of which complex structures consist. At yet another level, living molecules, which is to say organisms, become the objects of analysis. This is the realm of “analytic” molecular biology research, which traces the behavior of organisms back to their inner molecular genetic structures. At each level, analytic knowledge allows one to intervene technologically, alter the objects in question and devise novel objects. This is the “synthetic” side of each analytic science. In chemistry, for example, naturally occurring compounds such as sugar or vitamins can be produced synthetically and, in addition, compounds not known from nature, such as plastics, can be synthesized. Synthetic biology, it is expected, will lead to similar developments with regard to organisms. Synthetic biologists will be able to rebuild naturally occurring organisms and to create novel organisms by means of DNA synthesis (Kastenhofer 2013).

This straightforward account of scientific progress, and the place of synthetic biology within it, is closely connected to an ontological assumption. If explaining complex molecules amounts to knowing the simple molecules contained within the complex structure, and if explaining the function of an organism is tantamount to identifying and analyzing the genetic structure of this organism, then it is obviously assumed that the functioning of a complex object is the result of the laws and regularities governing the behavior of this object’s parts. As a correlate, if one knows the parts of an object and their functions, one can reliably predict the overall behavior of the object in question. This ontological assumption is the link to the machine metaphor. With regard to machines, the very same principles of part-whole relations and predictability are at work in reverse order. When one designs and assembles a machine, the idea is to combine parts with reliable and predictable functions in order to come up with a complex result that, in turn, and due to the reliability of its parts, fulfills specific functions and purposes in reliable and predictable ways. Consequentially, if what one does in synthetic biology is correctly captured by the account given above, the objects of one’s research must be thought to share important ontological aspects with those entities that one calls “machines”. If one transfers the relevant characteristics of the machine paradigm to the realm of synthetic biology and single cell organisms, this, then, is the resulting list: Firstly, the behavior of a single cell organism is to be explained by reference to its molecular, genetic parts. Thus, single cell organisms can be designed and can be built part by part. Secondly, designing and building an organism entrenches a specific function in the organism. This function fulfills, from the perspective of the designer, a specific purpose. Thirdly, the resulting organism’s behavior can be predicted reliably given sufficient knowledge of its parts (Boldt 2013).

## Envisaging potential environmental adverse effects

When one intends to build a reliable object with specific functions, and when one assumes that the reliability and predictability of the object is a result of the reliability and predictability of the behavior of its parts, a natural point to start will be to analyze these parts. As soon as one can assume to have sufficient knowledge about the parts, determining the behavior of the object must appear to be, in principle, a matter of computation. In turn, if the object behaves in unpredicted ways, leading to unintended side effects, the cause of the failure must be assumed to be located in the inner make-up of the object. Now, on the one hand, if one imagines this object to be, for example, an electronic device, this perspective covers adequately the demands of building safe machines and of dealing with side effects, one may assume. On the other hand, if one imagines the object to be an organism, there are good reasons to doubt the adequacy of this outlook.

For one thing, organisms evolve. Evolution is a process involving chance mutations within a genome. Chance mutation is, by definition, an unpredictable process. This counteracts any attempt to design a stable, reliable organism that preserves its design function from generation to generation. Evolutionary change thus is a main obstacle to building organisms the behavior of which can be predicted over long periods of time. For example, it has been proposed to insert toxin producing genetic circuits into genetically modified organisms. Under normal conditions the genetic circuits are repressed and do not express the toxin. If one observes that the organism proliferates in unintended ways, or leaves its assigned host environment, one can activate the gene circuit by adding a metabolite, for example. Then, toxin is produced and the microbes die. However, as experience shows, these “suicide genes” are prone to undergo mutation over time, which compromises their functionality (Wright, Stan, and Ellis 2013).

For another thing, organisms interact in manifold ways with each other and their environment. In microbial multispecies communities, bacteria influence each other via direct physical contact, metabolic interdependencies and coordinative signaling systems, thus maintaining ecosystem equilibrium (Guo, He, and Shi 2014). With regard to microbial communities, this modelling requires close ongoing collaboration between theorists and experimentalists (Zaccaria, Dedrick, and Momeni 2017). Modelling a multispecies ecosystem not restricted to microbes obviously would be an even greater challenge. On the one hand, this is not much of a surprise for molecular biology research practice. On the other hand, if one confines oneself to the machine paradigm, these multi-level interactions must come unexpectedly. In order to build a reliable machine one would restrict the number of external factors that can influence the workings of the machine to those that are necessary for the machine to fulfill its design purpose. If organisms are viewed as machines, thus, one would not expect them to interact in these multiple ways with the environment. This may lead to underestimating possible adverse effects of genetically altered organisms in the environment, since on the one hand they are products of synthetic biology and described as machines, on the other hand, they are still natural living beings.

As an aside, in addition to chance mutation and ecosystems interactions, reproduction and growth challenge ideals of the machine perspective as well. Besides predictability and reliability, machines are supposed to be entities that efficiently fulfill their functions. Up to a certain point, growth and reproduction are prerequisites for microbes to produce a specific valuable metabolite, for example. Above this limit, though, growth and reproduction take up energy that otherwise could be used in the metabolic process to produce larger amounts of the required substance. Life’s tendency to preserve itself both with respect to individuals and with respect to generations thus at a certain point counteracts attempts to utilize organisms as efficient production facilities. Therefore, optimizing organisms in this respect would be tantamount to devising non-living biological molecular networks, such as genetic cell-free production systems.

Turning back to safety related characteristics of natural organisms that are difficult to incorporate into the machine paradigm, it is worth noting that evolutionary genetic changes are small, stepwise changes. Mutagenesis is a selective alteration of the genome, and it builds upon the design as it has evolved up to that point, regardless of whether a novel design may fit an ecological niche better than existing organisms. Now, at first sight the machine metaphor does not seem to prompt engineering radically genetically altered organisms rather than sticking more closely to known ones. After all, if natural organisms can be explained in terms of the functioning of a machine, building a machine might just as well consist of rebuilding a slightly altered natural template. On a wider understanding, though, taking into account the content of the machine metaphor regarding parts-whole relations and concepts of scientific progress, confining oneself to re-designing natural organisms must appear to be a gratuitous restriction of one’s scientific and technological abilities. Rebuilding natural organisms may be a first step to underpin the claim that one has accurately analyzed and explained the functioning of organisms. The ultimate proof of this claim, though, is designing and engineering organisms that are not known in nature. Similarly, to forego exploring potential benefits that only novel organisms may possess would appear to be an imprudent confinement to the well-trodden paths of nature, a confinement that one cannot justify scientifically or technologically from the machine point of view, once sufficient knowledge of genetic parts is available. Thus, this striving towards novelty is fueled by epistemological reasons and by reasons pertaining to exploring uncharted terrains of organismic functions and their possible benefits. Reflections on safety are no inherent part of this perspective, but must be added from the outside, as it were. Now, taken to the extreme, designing novel genetic organisms may include organisms with DNA made from alternative amino-acid systems. Given these xenobiological organisms’ reduced abilities to interact with natural organisms, they may in some cases offer safety advantages (Schmidt 2010). Nonetheless, as a general rule, the more radical an organisms differs genetically from natural organisms, the less one can predict its environmental effects, including adverse effects. As the example of invasive species, novel organisms to the invaded habitat, shows, these risks do exist.

To sum up, chance mutation and evolutionary change, and, in addition, organism-environment interactions are characteristics of organisms that challenge attempts to predict their behavior reliably. Mutations modify the internal genetic structure of an organism, potentially leading to altered functions, and they occur, qua being chance mutations, unpredictably. Organism-environment interactions involve high numbers of variables and values rendering attempts to model the development of multispecies ecosystems over time challenging. From the machine point of view, characteristics of organisms such as mutagenesis and multi-level environmental interactions come unexpectedly. In order to build a reliable machine one would want to avoid any chance changes to the design that have an effect on the function of the machine, and one would want to restrict the number of external factors that can influence the workings of the machine to those that are necessary for the machine to fulfill its purpose. Thus, if one restricts one’s perspective to the machine paradigm, one may overlook potential risks and side effects when a genetically altered organism, viewed as a machine, is engineered for an application in the environment. Finally, the ontological assumptions inherent to the machine metaphor render engineering genetically radically altered, and in this sense novel organisms the ultimate proof of success with regard to explaining organisms. By its own logic, this striving does not anticipate environmental safety risks.

Claiming that synthetic biology views organisms through the lens of the machine paradigm, and arguing that the machine paradigm tends to downplay evolutionary change and interaction within ecosystems does not necessarily entail calling for a replacement of the machine metaphor. The machine paradigm may be helpful in many respects when one analyzes genetic structures and develops novel gene networks. It does entail though that when it comes to applications in the environment, supplementary expertise is needed to assess environmental safety risks. This expertise needs to include a focus on evolutionary change and ecosystems interactions, which is to say that it needs to include expertise in ecosystems biology, evolutionary biology, and developmental biology. This is, of course, no news for existing safety assessments of genetically engineered organisms for environmental use. These and other multidisciplinary safety assessment procedures already do exist. Being aware of the possible impact of the machine metaphor on safety perception backs up and reinforces these practices.

One special issue deserves mentioning here. Mutagenesis and novelty differ with regard to safety assessment challenges. To start with, mutagenesis renders the safety assessment of any engineered organism difficult. Nonetheless, in the case of engineered organisms that are slightly modified versions of a natural template, risk assessment can rely on experience with this template. In contrast, releasing a novel synthetic organism into the environment raises safety concerns because the effects of this organism as it is, prior to any genetic mutation, on existing ecosystems is hard to assess. After all, in this case there is no single natural template, which could serve as a basis for risk assessment. This second challenge may actually render plausible a demand to refrain from releasing synthetic organisms at all. This demand, though, is warranted only in the case of radically altered organisms. One could imagine that introducing novel synthetic organisms stepwise, starting from a non-radical version of the synthetic organism via ever more radical designs, could help to deal with this problem adequately, if this procedure allows enough time to get acquainted with each version of the organism and its environmental effects.

## Machines and moral agents

As long as synthetic biology is confined to genetically engineering single-cell organisms, discussing issues of moral status may appear superficial. What is more, scenarios of synthetic biology modifications of mammals and humans in the future mention, for example, reconstructing the mammoth by way of modifying elephant germ cells, and genetically engineering the human germline in order to improve the immune system (Church and Regis 2012). Since modifications of this kind leave all those capacities of animals and humans intact that are essential for their moral status, there would be no reason to suppose that ascriptions of moral status to the mammoth, or to the genetically altered human, would differ from ascriptions of moral status to elephants or non-genetically-altered humans, it seems. What would change, though, when synthetic biology turns to multi-cell organisms, mammals, and humans, is that, following the machine metaphor, an understanding of animal and human behavior is imported that may stand in tension with and thus may weaken understandings of these behaviors that lend credibility to ascriptions of moral status.

Many ethical theories, among them utilitarianism and deontolgy, agree that a necessary condition for moral agency is the capacity to distance oneself from one’s interests, and to reflect upon these motivations from an evaluative point of view that takes into account well-being, interests, or rights of others. In utilitarianism, for example, this reflective distancing enables an evaluation of possible actions in terms of the effects of the consequences of these actions on the interests of others that are affected by the actions. In Kantian deontology, the distancing comprises evaluating whether the maxim of one’s envisaged action (i.e., the statement that one wants to perform an action of a certain type in a certain situation in order to attain a specific end) could be upheld in a hypothetical world in which all moral agents would follow this maxim.

The ability to distance oneself from the interests that would otherwise lead one to act in a certain way is not easily reconcilable with the machine paradigm. To begin with, this ability appears to include the ability to prove any prediction of one’s future behavior wrong simply by acting differently. If one is confronted with a prediction of one’s behavior or if one has a suspicion that someone wants to take advantage of a certain regularity of one’s past behavior that he reckons will continue in the future, one can change one’s behavior and act contrary to these expectations, if one has an interest in not being predictable. What is more, suspending one’s actions and reflecting upon them appear to be best understood as an interpretive activity. Determining interests or maxims involves determining actions that someone, following his interests or maxims, respectively, is prone to perform. As Alisdair MacIntyre, among many others, has argued, actions are not entities that can be perceived immediately as what they are. Their identity depends on the ends they are supposed to serve. These ends can be manifold and link up to larger narratives about who the agent is and what kind of life she leads. For example, whether what I am doing is mowing the lawn, exercising, doing a favor to my wife, or irritating my neighbor cannot be discerned by looking at my movements. Understanding my action presupposes knowledge about me, my relations, my habits, and my plans (MacIntyre 2013, 237–263). In turn, such knowledge presupposes knowledge about what it is like to experience emotions, to be in relation with others, and potentially to make sense of and find meaning in life as a whole. When attempting to discern what an action is, one thus needs to interpret the events in question in terms of different narratives that make sense of them and picking the one that appears to fit best to this specific and other of my actions. Looking backward at past actions, these narratives help to understand what one actually did and why. At the same time, looking forward, these narratives shape how one will act in the future. They render certain options more meaningful than others, and thus lead one to act in certain ways.

Now, let us suppose one puts together a robot equipped with artificial intelligence of a kind that allows the robot to, one, change its behavior when it is confronted with predictions of it. Two, the robot continually engages in reflecting about narratives that make sense of its actions, and applies these narratives to shape its future behavior. Three, these narratives are based on knowledge of what it is like to experience emotions, to be in relation with others, and to make sense of life as a whole. As it seems, in this case there would be no reason not to ascribe the status of a moral agent to this robot, unless, and I am following a realist supposition on this issue here, it turns out that the robot does not really possess these properties but somehow mimics them (Torrance 2014).

At the same time, though, would there be good reason to call this robot a machine, that is to say, an object the behavior of which can be explained in terms of parts that are effectively arranged in order to attain specific behavioral ends? Given that this robot can stop doing what it is designed to do when it is told about its design ends, and given that it continually shapes and reshapes its ends and its future by interpreting its actions in terms of larger narratives about what is important and lends meaning to its life, this does not appear plausible. While at the micro-level of bit processing and electronic limb control, this robot may well be described as a machine, at the macro-level this description would not fit any more.

In much the same way, while isolated processes at the level of molecular interactions in the human body may be comparable to machines in meaningful ways, humans as acting and reflecting whole organisms capable of moral agency are not. Applying the machine metaphor to humans would amount to calling into question the reality of the ability to distance oneself from one’s interests, to prove wrong predictions of one’s behavior, and to shape one’s future according to larger human life narratives. Since these are conditions for moral agency, applying the machine metaphor to humans would amount to calling into question the reality of the human ability for moral agency. One may compare this line of thought to the genetic determinism assumption. According to this assumption, DNA is a blueprint that determines phenotype traits and behavior. It was argued further that since phenotype human traits and behavior are determined by genetic make-up, the genotype is the actual agent behind human behavior, rendering beliefs in human moral agency an illusion (Dar-Nimrod and Heine 2011). The machine metaphor is well suited for similar claims since its explanatory scheme of parts reliably causing behavioral effects of the entity as a whole does not leave room for those explanatory schemes that sustain ascriptions of moral agency.

## Machines and moral patients

Engineering human DNA by way of synthetic biology circuit design is still science fiction, even though research approaches to alter genetic circuits in mammalian cells to prevent disease are already explored (Black, Perez-Pinera, and Gersbach 2017). Envisaging synthetic biology multi-cell organisms, including insects, is much closer to reality, though (Markson and Elowitz 2014). Now, since animals do not possess the status of moral agents, applying the machine metaphor to them cannot undermine their moral status in this respect. Both according to everyday attitudes and according to many ethical theories, though, some animals have moral status in the sense that protecting their well-being is a moral obligation, and inflicting harm on these animals constitutes a prima facie moral wrong that needs to be justified by other, overriding ethical obligations in order to be acceptable. That is to say, some animals can be said to possess the status of moral patients. The question then is whether applying the machine metaphor to animals may undermine practices of ascribing the status of being a moral patient to them.

In animal ethics, different sets of traits are identified as necessary conditions for being a moral patient. Being able to experience suffering and enjoyment, for example, is an intuitively appealing first and basic candidate. These experiences are not neutral sensations but involve an element of evaluation. Experiencing pain and joy is bound up with needs, desires, interests, or, as an overarching term, attitudes (Basl 2014). All of these concepts suggest the idea that animal behavior of the relevant kind must be understood as being stretched out between a perceived actual state of being and an anticipated future state of being. Metaphorically speaking, unlike causal descriptions, descriptions in terms of desires, interests, and attitudes suggest that chains of behavior are not pushed forward by starting conditions according to laws of animal behavior, but are due to pulling forces, namely the attractiveness or unattractiveness of different possible future state of affairs. Weighing these courses of action according to what matters for the animal introduces an element of human-like agency that is captured in Tom Regan’s concept of being “the subject of a life” (Regan 1983). If this is a correct understanding of moral patiency, being a moral patient and being a moral agent can be interpreted as ascriptions to organisms along a line of an increasing ability to take a stance towards one’s motivational states. Avoiding pain, seeking pleasure, having needs, having desires, following interests, and shaping one’s life according to narratives may be understood as steps along this line of an increasing ability to suspend immediate impulses to act and to shape one’s future in accordance with evaluative reflection (Jonas 2001).

Again, then, the capacity to be the subject of a life stands in tension to the machine paradigms assumption that the behavior of an object is to be explained by internal parts that effectively, reliably and predictably cause this behavior. Applying the machine metaphor to animals that possess the status of moral patients undermines these moral status ascriptions since it calls into question the assumption that the behavior of an animal can be a basic form of agency of a subject of a life.

As a consequence, while applying the machine metaphor to genetic processes in and between human and animal cells may help to accumulate technologically useful knowledge about these processes, applying it to animals and humans as whole organisms does not do justice to the characteristics of animal behavior and human agency and renders ascriptions of moral status unintelligible.

## Spearhead science and technology

The machine metaphor invokes an account of scientific progress according to which the behavior of any complex object can be explained by reference to its parts. A complete explanation of reality thus starts from its most basic parts, moves on via intermediate ones, and ends with explaining highly developed organisms and human beings. In principle, the interactions of objects at each level are supposed to be contained in theses explanations, since they can be seen as a subset of an object’s individual behavior. If one strictly adheres to this paradigm, explanations in terms of, for example, teleological concepts, or folk psychology, or narrative accounts of historical events, are valid only if they can be shown to conform to physiological, chemical and physical explanations. The latter explanations, then, appear as “real” explanations, whereas the former ones at best are shortcuts for those real ones.

From the point of view of the machine paradigm, the preference for explanations in terms of parts is a preference for explanations that refer to causal laws or law-like regularities, since only regular behavior allows for reliable predictions, which is what one needs if one wants to build a machine. Teleological and narrative accounts of events, by contrast, are accompanied by ambiguity and uncertainty. A person’s intention, for example, cannot be deduced directly from her action as a physical, observable event, but depends on the larger framework of the person’s long-term plans, professional aims, private aspirations, situational inclinations, and many other attitudes, character traits and objectives. Any one of these features can play a role in grasping why the person acted in a certain way und thus in grasping her intention. Now, even if the person herself picks out one possible end and declares that her intention was to reach this rather than another end, doubt necessarily remains as to whether she is ultimately correct, and other convincing stories might be offered that shed new light on her behavior. Academic disciplines such as history and social and political science inevitably have to get along with these ambiguities and uncertainties. By their own standards, this does not undermine claims to knowledge and expertise. From the machine point of view, though, these claims to knowledge must remain dubious.

What is more, following the machine paradigm and its implications for scientific progress, synthetic biology can be singled out among the natural sciences with regard to its state of development. While physics has already reached a level of analytic accuracy that enables, among many other things, the production of novel synthetic elements, and while chemistry has already analyzed chemical reactions up to a point where it becomes possible to synthesize novel compounds such as plastics, biology now has advanced to precisely the threshold where purposeful designing, arranging and rearranging of genetic parts becomes possible to such a degree that the resulting genetically engineered organisms can be regarded as “novel”. This implies that right now synthetic biology is to be regarded as the spearhead of scientific and technological progress. From its own perspective, it is a discipline that is about to develop the most accurate knowledge about organisms up to date, and it is about to turn this knowledge into reliable technological means to modify and control behavior (Bensaude Vincent 2013).

Now, many of the serious and unresolved problems societies grapple with involve organisms. To name but a few, food has to be provided for a growing population. Crop plants are unable to grow in large areas of the earth due to drought. Oil spill threatens marine ecosystems. All of these problems are well known, and obviously so far bringing to bear the available technological tools on these problems has not helped to solve them, nor have social and political measures had decisive effect. If one is to look for new solutions, following the machine paradigm one will in this situation first of all place one’s hopes on technology rather than social and political measures, since the latter cannot, as it is assumed, be informed and guided by truly reliable knowledge. What is more, since available technologies have been utilized to no avail, it will seem natural to turn to the latest significant development in this field. Given the machine paradigms background story, synthetic biology thus can come to be regarded as not merely one among other, but rather the foremost candidate for solving societal and environmental problems.

Critics of what has come to be known as “technological fixes” to societal problems have long argued that these approaches suffer from a variety of drawbacks (ETC 2007). For instance, while for-profit companies in rich parts of the world may develop these technologies, the site of application and the end users may be located in low-income countries. Technological solutions thus may contribute to global inequality and entrench economic dependency. What is more, high-tech applications may fail to provide sustainable, long-term solutions, since local knowledge and resources may not suffice to repair the application and keep it functional. Consequently, opting for low-tech solutions, or alternatives to technical solutions, or changing societal conditions, for example regarding education and training, may often promise better outcome than high-tech applications. Owing to the machine metaphor and its wider implications, one may tend to overestimate the share synthetic biology applications may contribute to solving societal problems.

Again, this is not an argument for abandoning the machine metaphor, nor is it an argument to renounce developing synthetic biology applications and assessing their potential benefits. Rather, it is an argument for staying realistic with regard to synthetic biology hopes and promises, for keeping track of the whole field of possible technological and social solutions to societal problems, and for embedding synthetic biology applications in a social context that allows long-term safe and just use.

## Conclusion

The machine metaphor in synthetic biology is a powerful conceptual lens. It can fruitfully lead research to analyze the relation of genetic parts to the functions of the organism as a whole and to determine ways to engineer organisms. At the same time, firstly, the machine metaphor systematically fades down characteristics of organisms such as evolutionary development and ecosystems interactions. This may lead to underestimating potential environmental side effects of synthetic organisms. Moreover, from the point of view of the machine metaphor, confining oneself to building slightly altered natural organisms falls short of exploiting the full potential of synthetic biology, which from this point of view lies in its ability to engineer novel organisms, such as organisms containing synthetic DNA parts taken from a variety of natural templates, and organisms containing DNA based on alternative base pairs. How to assess possible side effects of novel synthetic organisms on the environment is an open question, though. Secondly, from the perspective of the machine metaphor, viewing an entity as a moral agent or patient, respectively, becomes dubious. From this perspective, the characteristics of living beings that warrant ascriptions of moral status must be reinterpreted as illusionary phenomena since they stand in tension to explanations in terms of parts that reliably bring about specific, predefined effects. Thirdly, the machine metaphor contains a concept of scientific progress and synthetic biology’s place within this progress. This concept stresses some aspects of the nature and potential of science and technology in general and synthetic biology in particular, and hides others. Inherent in this perspective is a tendency to overestimate the potential contribution of synthetic biology applications to solving societal and environmental problems.

Being aware of the implications of the machine paradigm can help to include supplements to this perspective that overcome its ethical limitations and to reinforce practices that already mitigate these effects. With regard to environmental safety, awareness of the force of the machine metaphor thus backs up practices of multi-disciplinary safety assessments. Building novel organisms stepwise may be another measure to mitigate risks. With regard to the potential moral status of organisms, awareness of the explanatory scheme implicit in the machine metaphor helps to understand that ascriptions of moral status hinge on characteristics of animal and human behavior that are out of the reach of the machine metaphor. Thirdly, and finally, with regard to assessing means to solve societal problems, awareness of the internal logic of the machine metaphor and its epistemic background story may safeguard against overestimating the capacities of synthetic biology to successfully deal with these challenges.
